# Supplementary material for: Compressive stress-mediated p38 activation required for ERα + phenotype in breast cancer
Source: Nat Commun. 2021 Nov 29;12:6967. doi: 10.1038/s41467-021-27220-9 (PMC8630031; doi:10.1038/s41467-021-27220-9)
Supplement: Supplementary file 1 — Supplementary Information [file 41467_2021_27220_MOESM1_ESM.pdf]

Supplementary Information

Compressive Stress-Mediated p38 Activation Required for ERα+ Phenotype in Breast Cancer

Supplementary Figure 1.

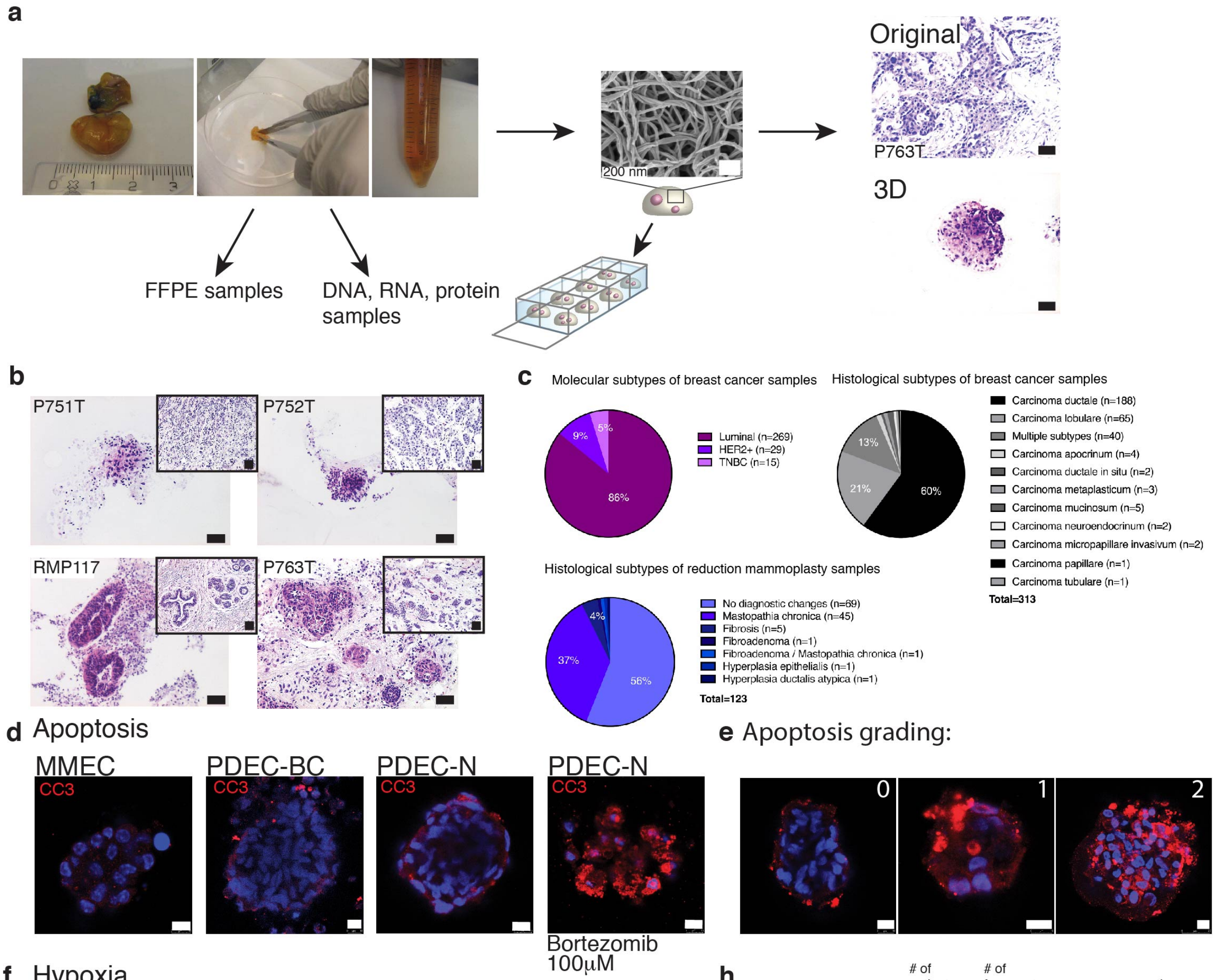

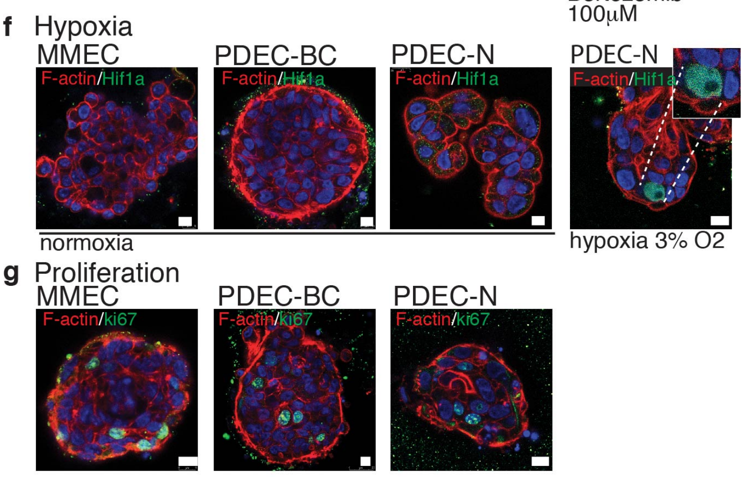

h

		# of patients	# of fragments	average	time
Hypoxia (Hif1a)					
	PDEC-N	3	10	nd	7d
	PDEC-BC	3	10	nd	7d
	PDEC-N *	2	8	53 %	7d
Proliferation (ki67)					
	PDEC-N	7	32	45 %	7d
	PDEC-BC	4	20	42 %	7d
Apoptosis (CC3)					
	PDEC-N	3	28	96 %=0	7d
	PDEC-BC	3	39	79 %=0	7d
Size					
	PDEC-N	11	110	97 µm	7d
	PDEC-BC	14	128	89 µm	7d

*3% oxygen

i

Patient:	Gene:	Variant:	Type:	GQX:	Frequence:	Depth:	dbSNP ID:
P9T primary tumor	TP53	G>C/C	snv	100	99.7	3625	rs1042522
P9T PDEC-BC 7d	TP53	G>C/C	snv	100	99.74	4683	rs1042522
P13T primary tumor	TP53	G>G/C	snv		52.16	3574	rs1042522
P13T PDEC-BC 7d	TP53	G>G/C	snv		48.5	3470	rs1042522
P15T primary tumor	TP53	G>G/A	snv	100	55.3	5000	rs121913343
	TP53	G>G/C	snv	100	77.32	3303	rs1042522
P15T PDEC-BC 7d	TP53	G>G/A	snv	100	78.43	3968	rs121913343
	TP53	G>G/C	snv	100	88.97	2610	rs1042522

j

Patient #	ERα+ original	ERα+ 7d
P88T	3	0
P103T	3	0
P104T	3	0
P130T	3	0
P131T	3	0
P132T	3	1
P134T	3	1
P140T	3	0
P147T	0	0
P218T	3	0
P219T	3	0
P224T	3	0
P225T	3	0
P236T	3	0
P300T	0	0
P660T	0	0
P646T	3	0
P647T	3	0
P648T	3	1
P661T	0	0
P679T	3	1
P680T	3	0
P682T	3	1
P737T	3	1
P756T	3	1

Supplementary Figure 1. Patient-Derived Explant Culture (PDEC) Platform. a, Sample processing. Breast epithelial tissue samples from reduction mammoplasties or breast cancer samples from elective surgeries were brought to the tissue culture laboratory within hours of surgery. The sample was divided in three parts. One part of the tumor was stored as a formalin-fixed paraffin embedded (FFPE) sample and another part was snap-frozen for protein/DNA/RNA profiling at -80 °C. The remaining part was enzymatically dissociated into small fragments, which were embedded in 3D matrix. Images on the right show histological sections with H&E staining of the original and 3D cultured explant. **b**, H&E-stained histological sections of PDEC-BC and PDEC-N samples, which were cultured in a 3D culture for 7 days. Control is the corresponding uncultured sample (n= 3 biologically independent samples). **c**, Molecular and histological subtypes of the breast cancers and histology of reduction mammoplasties used in this study. **d**, Immunofluorescent images of MMEC, PDEC-BC, and PDEC-N cultured in 3D matrix for 7 days and stained for apoptosis marker cleaved-caspase 3 (CC3). Bortezomib (100 μM) treatment was used as a positive control for apoptosis (n= 3 biologically independent samples). **e**, Immunofluorescent images as in **d** to illustrate the apoptosis grading (n= 3 biologically independent samples). **f**, Immunofluorescent staining of MMEC, PDEC-BC, and PDEC-N with hypoxia marker, Hif1α. PDEC-Ns grown in 3% oxygen were used as a positive control for hypoxia (n= 3 biologically independent samples). **g**, Immunofluorescent staining of MMEC, PDEC-BC, and PDEC-N with antibody specific for proliferation marker ki67 (n= 3 biologically independent samples). **h**, Quantification of apoptosis, proliferation, hypoxia, and explant sizes of PDEC-BC and PDEC-N (nd = not detected). N = independent experiments are listed. **i**, Tp53 mutation profiles of tumors from three patients (P9T, P13T, P15T) and the corresponding PDEC-BCs cultured in 3D for 7d. **j**, Quantification of ERα+ cells in 25 patient samples either uncultured or cultured in BMx-Mat for 7 days. Grade 0 = <10% ERα+, Grade 1 = 10-39% ERα+, Grade 2 = 40-69% ERα+, Grade 3 = 70-100% ERα+. Scale bar = 10 μm.

Supplementary Table 1.
Sample preparation methods and the parameters for the rheological measurements

a

Matrix	Sample preparation method	Coating	Acceleration voltage (kV)
Egg white	Liquid propane freeze-drying	30 mA 120s platinium	1.5
GrowDex	Liquid propane freeze-drying	4 nm iridium	1.5
Agarose	Liquid propane freeze-drying	5 nm iridium	1.5
Alginate	Critical point drying	10 nm iridium	1.5
Alginate-RGD	Biological sample preparation	16 nm iridium	1
Matrigel 3mg/ml	Biological sample preparation	11 nm iridium	1
Matrigel 8.8mg/ml	Biological sample preparation	16 nm iridium	1
Collagen	Biological sample preparation	5 nm iridium	1.5
Ovomucin	Biological sample preparation	16 nm iridium	1.5

b

Matrix	Concentration (mg/ml)	Strain (%)	Stress (Pa)	Frequency (rad/s)
Agarose	70	1	0.3	1
Agarose	30	1	0.3	1
Agarose	20	1	0.3	1
Agarose	10	1	0.3	1
Alginate	70	1	0.3	1
Alginate	40	10	0.3 or 0.15	1
Alginate-RGD	40	1 or 5 or 10	0.3 or 0.5	1
GrowDex	10	1	0.3	1
Matrigel	8.8	1	0.3	1
Matrigel	3	1	0.3	1
Collagen	8.7	1	0.3	1
Collagen	3	1	0.3	1
Collagen	1.5	1	0.3	1
Ovomucin	50	1	0.3	1
Egg white	-	1	0.3	1

Supplementary Table 1. a, Sample preparation methods, coatings and acceleration voltages of different matrices for scanning electron microscopy (SEM). **b**, Parameters of the rheological measurements. Strain, stress and frequency for different matrices and concentrations.

Supplementary Table 2.
Primer sequences used in this study

Name	Oligonucleotide sequences:
TSO	TSO: AAGCAGTGGTATCAACGCAGAGTGAATrGrGrG
SMART PCR primer:	AAGCAGTGGTATCAACGCAGAGT
P5 SMART primer:	AATGATACGGCGACCACCGAGATCTACACGCCTGTCCGCGGAAGCAGTGGTATCAACGCAGAGT*A*C
Sequencing read 1:	GCCTGTCCGCGGAAGCAGTGGTATCAACGCAGAGTAC
**Name**	**The primer nucleotide sequences for mouse samples (5'-3') are as follows:**
PGR forward:	GTCACTATGGCGTGCTTACC
PGR reverse:	CCAGCCTGACAACACTTTCT
GREB1 forward:	CCATTTCCAGTGAGCCCATT
GREB1 reverse:	AGGTGCTTCTGTTTCTTGGG

Supplementary Figure 2.

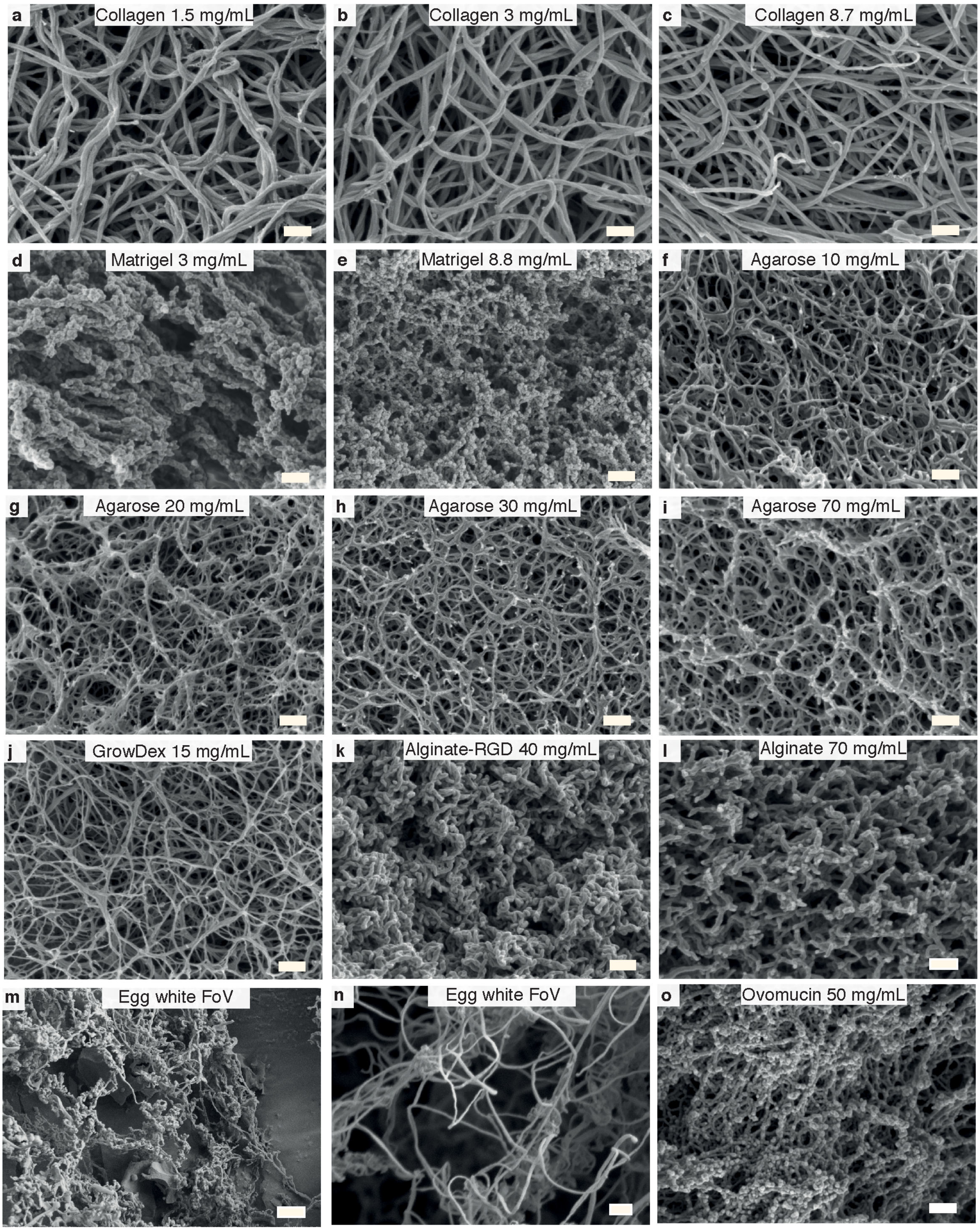

Supplementary Figure 2. Scanning Electron Microscopy (SEM) Images of the Matrices. **a**, Collagen 1.5 mg/mL. **b**, Collagen 3 mg/mL. **c**, Collagen 8.7 mg/mL. **d**, Matrigel 3 mg/mL. **e**, Matrigel 8.8 mg/mL. **f**, Agarose 10 mg/mL. **g**, Agarose 20 mg/mL. **h**, Agarose 30 mg/mL. **i**, Agarose 70 mg/mL. **j**, GrowDex 15 mg/mL. **k**, Alginate-RGD 40 mg/mL. **l**, Alginate 70 mg/mL. **m-n**, Egg white, two different fields of view (FoV) are presented to show the structural variation within a gel. **o**, Ovomucin 50 mg/mL. N = 3 independent samples per condition. Scale bar = 200 nm.

Supplementary Figure 3.

a

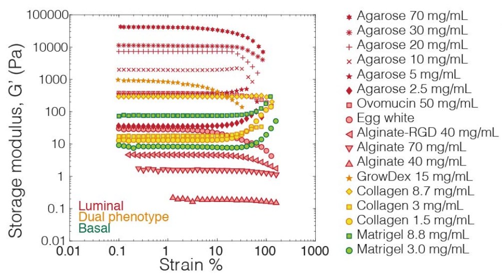

b

Matrix	Concentration c (mg/mL)	Storage modulus G´ (Pa)	Loss modulus G´´ (Pa)	Phase angle (degree)	Complex modulus G* (Pa)	Calculated elastic modulus E =2(1+v)G* (Pa)	#
Agarose	70	46000 ± 8000	2800 ± 200	3.5 ± 0.7	47000 ± 8000	130000 ± 30000	3
Agarose	30	14000 ± 4000	340 ± 30	1.5 ± 0.3	14000 ± 4000	39000 ± 10000	3
Agarose	20	8100 ± 700	183 ± 15	1.293 ± 0.004	8100 ± 700	23000± 2000	2
Agarose	10	1920 ± 140	45 ± 6	1.4 ± 0.1	1920 ± 140	5500 ± 400	3
Agarose	5	270 ± 80	7.0 ± 2.0	1.40 ± 0.03	270 ± 80	800 ± 300	3
Agarose	2.5	30 ± 8	0.9 ± 0.2	1.8 ± 0.3	30 ± 8	90 ± 30	3
Alginate	70	4.51 ± 0.14	7.5 ± 0.3	59.1 ± 0.8	8.8 ± 0.3	26.4 ± 0.8	3
Alginate	40	0.2 ± 0.1	0.9 ± 0.3	76 ± 5	1.0 ± 0.3	2.9 ± 0.8	3
Alginate-RGD	40	0.9 ± 0.6	8.0 ± 4.0	84 ± 3	8 ± 4	24.2 ± 9.5	3
GrowDex	15	900 ± 300	110 ± 50	7.1 ± 1.1	900 ± 300	2700 ± 700	3
Matrigel	8.8	76 ± 2	6.5 ± 0.2	4.94 ± 0.08	76 ± 2	227 ± 6	3
Matrigel	3	7.8 ± 1.0	1.3 ± 0.2	9.3 ± 0.3	7.9 ± 1.1	24 ± 3	3
Collagen	8.7	204 ± 15	31 ± 7	0.2 ± 0.0	210 ± 20	620 ± 50	3
Collagen	3	18 ± 4	3.2 ± 0.4	9.9 ± 1.4	19 ± 4	56.1 ± 9.7	4
Collagen	1.5	11 ± 2	1.5 ± 0.5	7.2 ± 1.5	11 ± 2	35 ± 6	6
Ovomucin	50	340 ± 30	39 ± 2	6.5 ± 0.4	350 ± 30	1040 ± 90	4
Egg white	-	40 ± 30	8.0 ± 5.0	12.2 ± 1.0	40 ± 30	110 ± 70	10

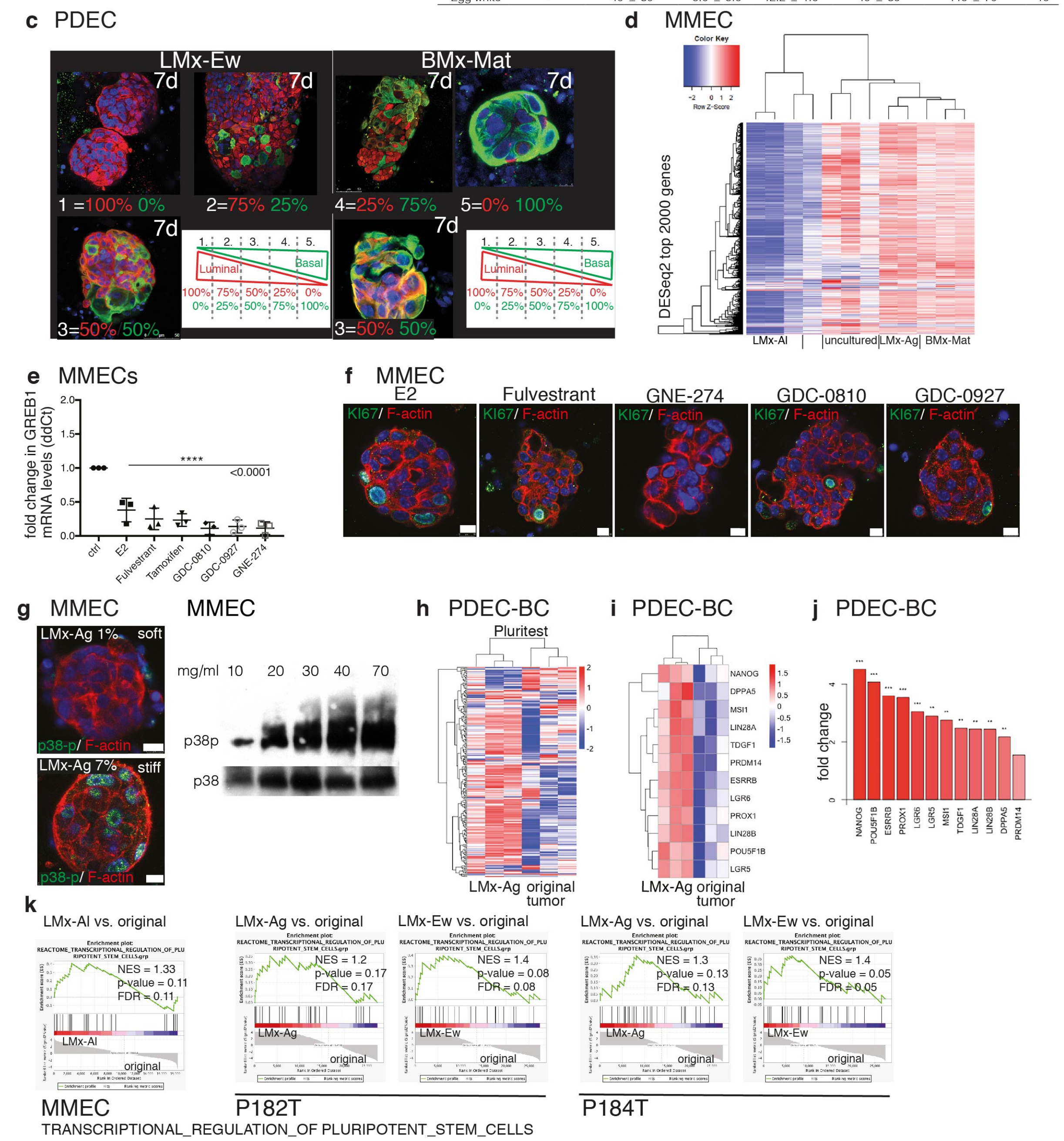

Supplementary Figure 3. Rheology, Cell Phenotype Quantification, Expression Profiles, and Antiestrogen Treatment Responses in Explant Cultures. a, The mechanical behavior of the matrices as a function of deformation (strain %) at +37 °C obtained from the oscillatory strain amplitude sweeps. LMx-type matrices (red) shows a large variation in the mechanical properties: from ultra-soft to stiff gels with a difference of five orders of magnitude and from strain softening (*G´* decreases as a function of strain %) to strain stiffening (*G´* increases as a function of strain %). From the mechanical point of view, Agarose 5 mg/mL, (0.5 %) Collagen 8.7 mg/mL, and Matrigel 8.8 mg/mL performed similarly. They had a storage modulus of ~ 100-500 Pa, which describes the stiffness of the gels. They all also showed strain stiffening, meaning that the material becomes stiffer and harder to deform when mechanical stress or strain is applied. **b**, Collected mechanical data of the matrices obtained from the oscillatory time sweeps at +37 °C. The elastic modulus (*E*) is estimated from the complex modulus (*G**) using the assumed Poisson's ratio of 0.44. N = independent experiments are listed. **c**, Quantification of luminal and basal identity in the explant cultures. Based on expression of luminal (CK8) and basal (CK14) markers, explants were assigned to one of five different groups; Group 1: 100% luminal; Group 2: 75% luminal, 25% basal; Group 3: equally positive for CK8 and CK14; Group 4: 75% basal and 25% luminal; and Group 5 :100% basal. Images show examples in each group. **d**, The LMx-Al gene expression of top 2000 differentially expressed genes. The expression profiles differ from LMx-Ag-, BMx-Mat-grow explants, and from their original uncultured sample in MMECs. The columns are clustered by sample and rows are clustered by gene. The dendrogram height indicates the distances between clusters in the gene expression profiles. **e**, QRT-PCR analysis of ERα target gene GREB1 mRNA levels in MMECs grown in 7d and treated with indicated compounds (E2 = 17β-estradiol). Statistical significance was tested using the one-way ANOVA test with Dunnett's multiple comparisons post hoc test: **** $p < 0.0001$. **f**, Immunofluorescence images of MMECs grown in LMx-Ag, treated with the indicated drugs for 24h and stained for Ki67 and F-actin. **g**, Immunofluorescence staining of p38p in a soft and stiff LMx-Ag matrix and western blot image of p38p expression in an increasing polymer concentration of LMx-Ag matrix (10 – 70 mg/mL). **h**, Enrichment of the pluripotency-related gene expression signatures in LMx-Ag-cultured PDEC-BC samples compared to the original tumors. The list of genes is adopted from PluriTest. **i**, The list of mammary epithelial-related pluripotency markers enriched in LMx-Ag-cultured PDEC-BC samples compared to the original tumors. **j**, The genes were ordered based on the fold change and differentially expressed genes are marked with an asterisk (adjusted p-value < 0.05*, 0.01** or 0.001***). **k**, Enrichment of the pluripotency-related gene set in the LMx-Al-cultured MMECs and in the LMx-Ew- and LMx-Ag-cultured PDECs. All data are presented as mean values +/- SD and n= 3 explants examined from 3 biologically independent samples. Scale bar = 10 μm.

Supplementary Figure 4.

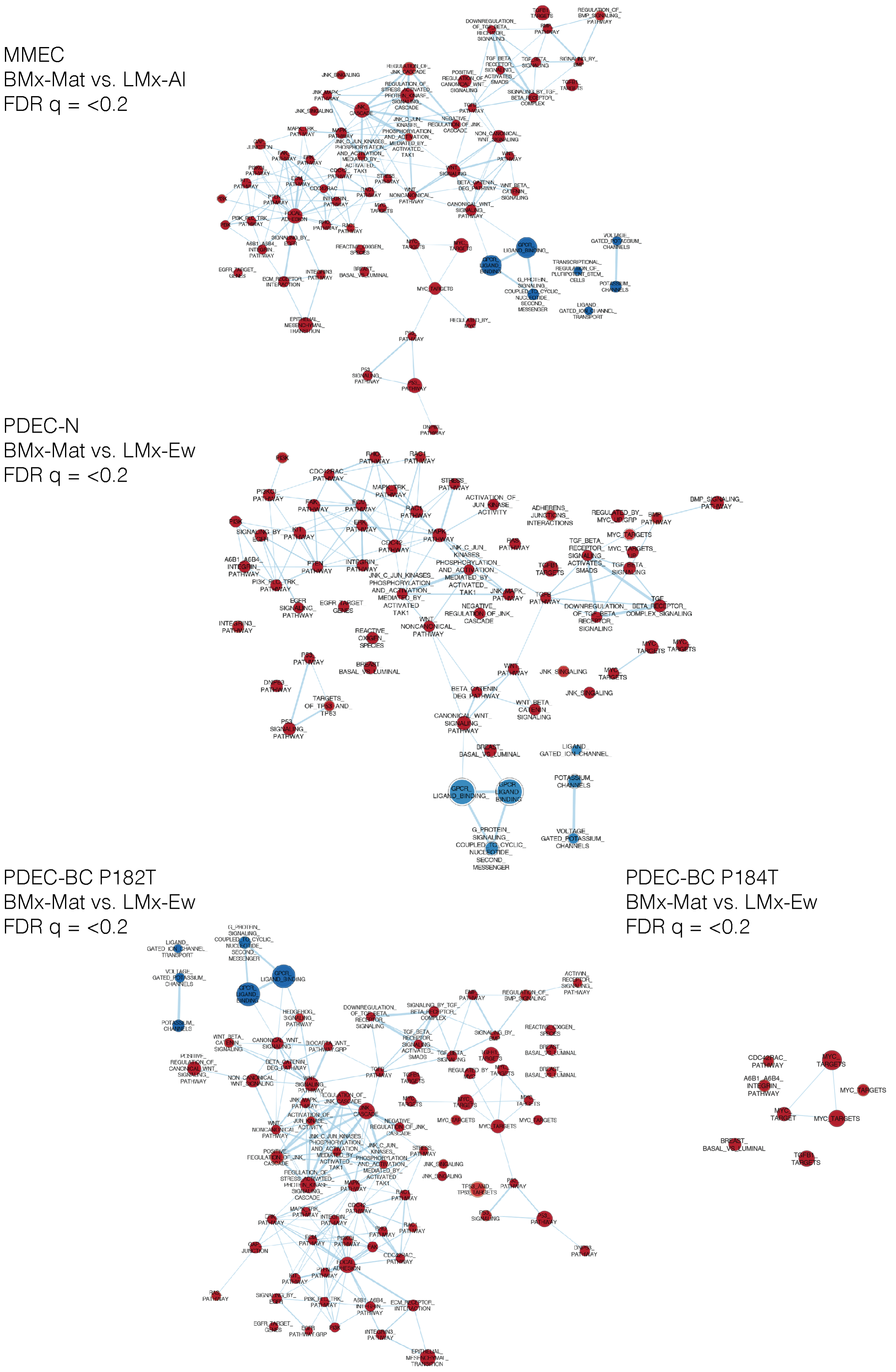

Supplementary Figure 4. Enrichment map of BMx-Mat enriched pathways. The Gene Set Enrichment Analysis indicates differences in the gene expression profiles between BMx- and LMx-gels. GSEA results are visualized using Cytoscape's Enrichment map. Node size: number of genes in the set; node color: red - enrichment in the BMx-Mat.

Supplementary Figure 5.

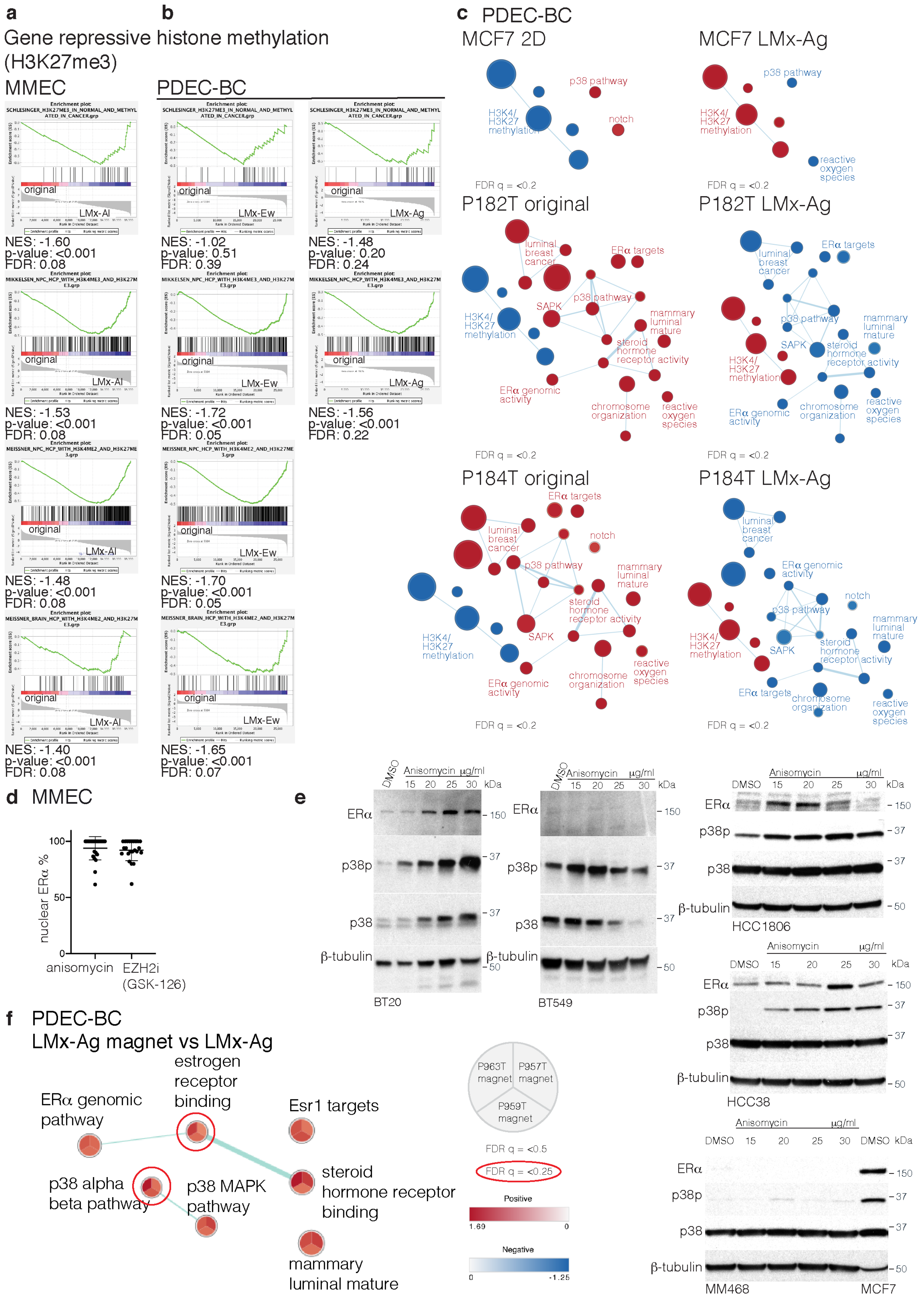

Supplementary Figure 5. Histone Methylation (H3K27me3) Patterns. **a**, GSEA analysis shows the enrichment of gene-repressive H3K27me3 signature in ERα- MMECs, cultured in LMx-Al. **b**, GSEA analysis shows the enrichment of H3K27me3 signature in ERα- PDEC-BCs, cultured in LMx-Ew and LMx-Ag. **c**, Enrichment map shows gene-set enrichment results of uncultured MCF7 and PDEC-BC (P182T, P184T) samples compared with LMx-Ag cultured explants and the vice versa. Node size, genes in pathway; node color, enrichment score. **d**, Quantification of the % of ERα+ cells of the total number of cells in MMECs grown in soft LMx-Al and treated with anisomycin and GSK-126 for 48h (n= 23 explants examined over 6 independent biological replicates in both conditions). Data are presented as mean values +/- SD. **e**, Western blot images show the effect of increasing concentration of anisomycin concentration on p38p/p38 and ERα expression in TNBC cell lines (n= 3). **f**, GSEA analysis shows the enrichment of p38 and ERα signaling related pathways in the magnet compressed LMx-Ag matrix compared to uncompressed samples. N = 3 biologically independent samples. Red circle shows the pathways that are enriched with FDR q = <0.25. Node size, genes in pathway; node color, enrichment score.

Supplementary Figure 6.

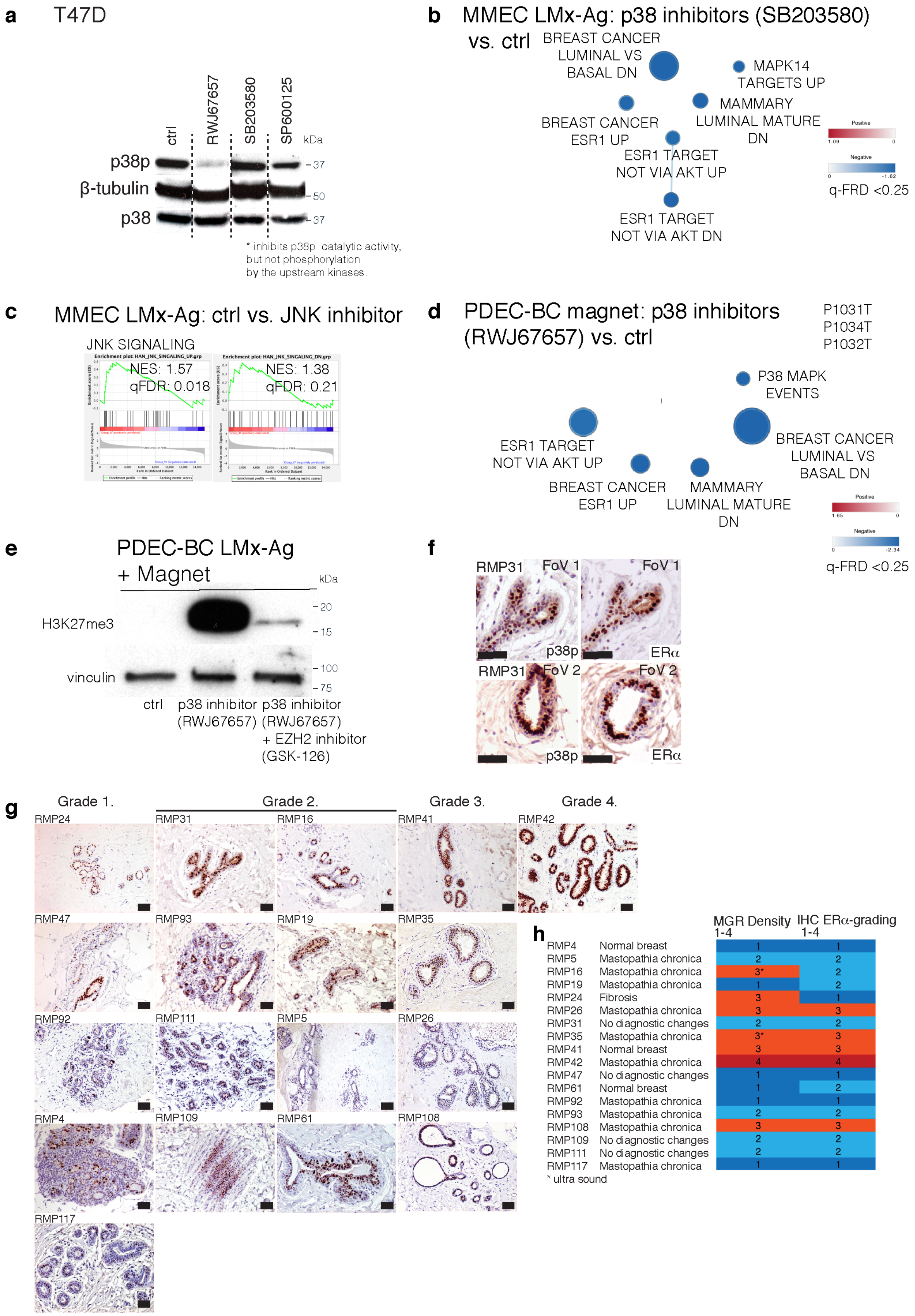

Supplementary Figure 6. Grading of the Mammographic Density and ERα. **a**, Two p38 MAPK inhibitors RWJ67657 and SB203580 were tested for their ability to suppress p38p. RWJ67657 specifically inhibits p38α with no effect on the p38γ, p38δ isoforms or other kinases[1]. We also used SB203580, which directly inhibits the p38 MAPK catalytic activity without interfering the upstream phosphorylation of p38p by the upstream kinases[2]. SP600125 is a JNK specific inhibitor (n= 3). **b**, GSEA analysis shows the downregulation of p38 and ERα signaling related pathways in the LMx-Ag matrix grown MMECs after p38 inhibition. Node size, genes in pathway; node color, enrichment score. **c**, GSEA analysis shows the downregulation of JNK pathway after JNK inhibition in LMx-Ag cultured MMECs. **d**, GSEA analysis shows the downregulation of p38 and ERα signaling related pathways in the magnetic compressed LMx-Ag matrix cultured PDEC-BCs from three different patients (P1031T, P1034T, P1032T) after p38 inhibition. Node size, genes in pathway; node color, enrichment score. **e**, Western blot shows the effect of p38 inhibitor (RWJ67657) with and without EZH2 inhibitor (GSK-126) on H3K27me3 (n= 3). **f**, Immunohistochemical staining of phospho-38 and ERα in normal breast epithelium (FoV = field of view) (n= 3 independent samples). **g**, Immunohistochemistry analyses of ERα expression in 18 reduction mammoplasty samples (n= 18). Each sample is categorized according to four groups (grades 1-4) according to the level of ERα+ expression (Fig 7 f). **h**, The table showing ERα+ expression (grade 0-4) and corresponding mammographic breast density values (MGR density 1-4). Patients diagnosed using ultrasound are indicated with an asterisk. Scale bar 50= μm.

Supplementary Figure 7.

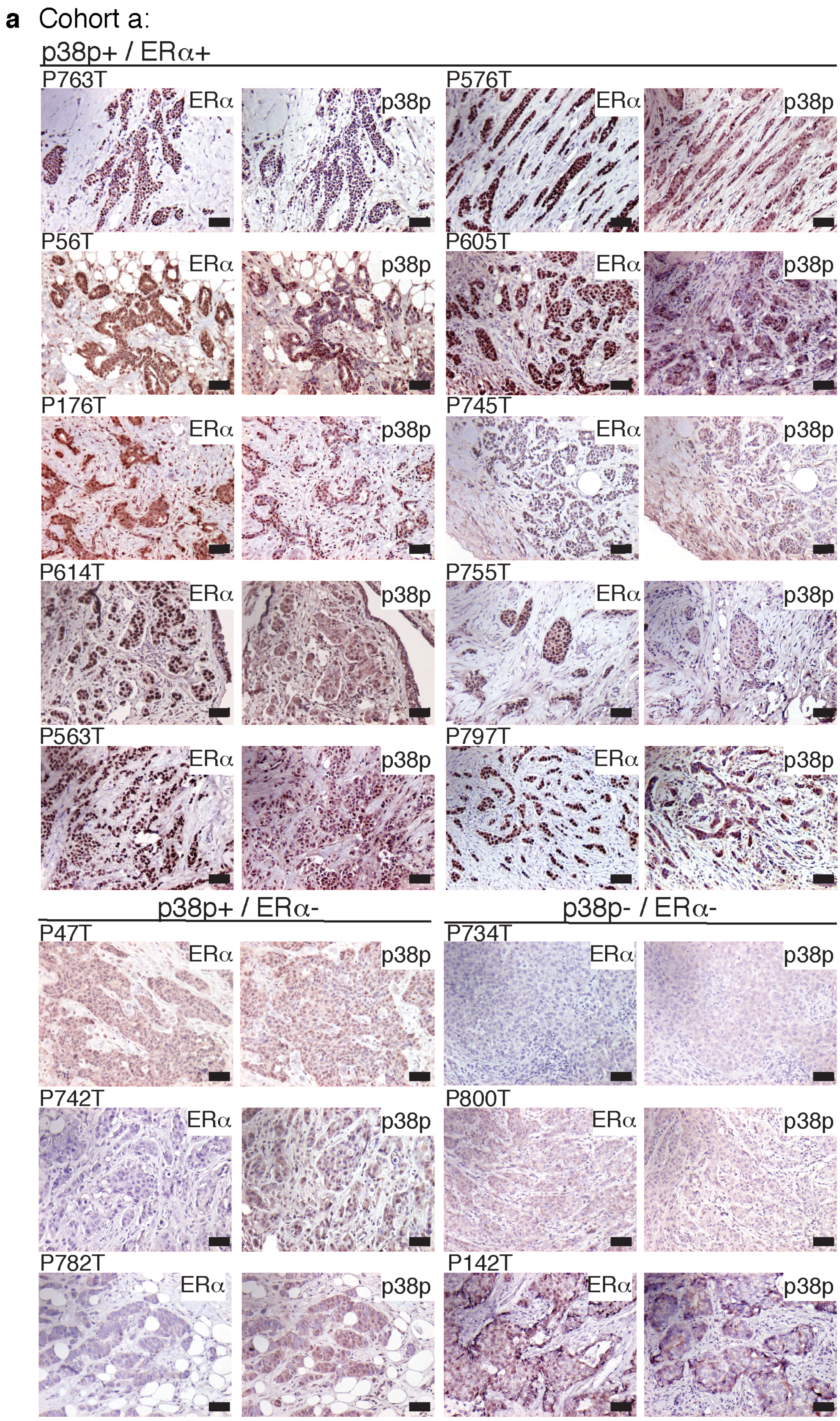

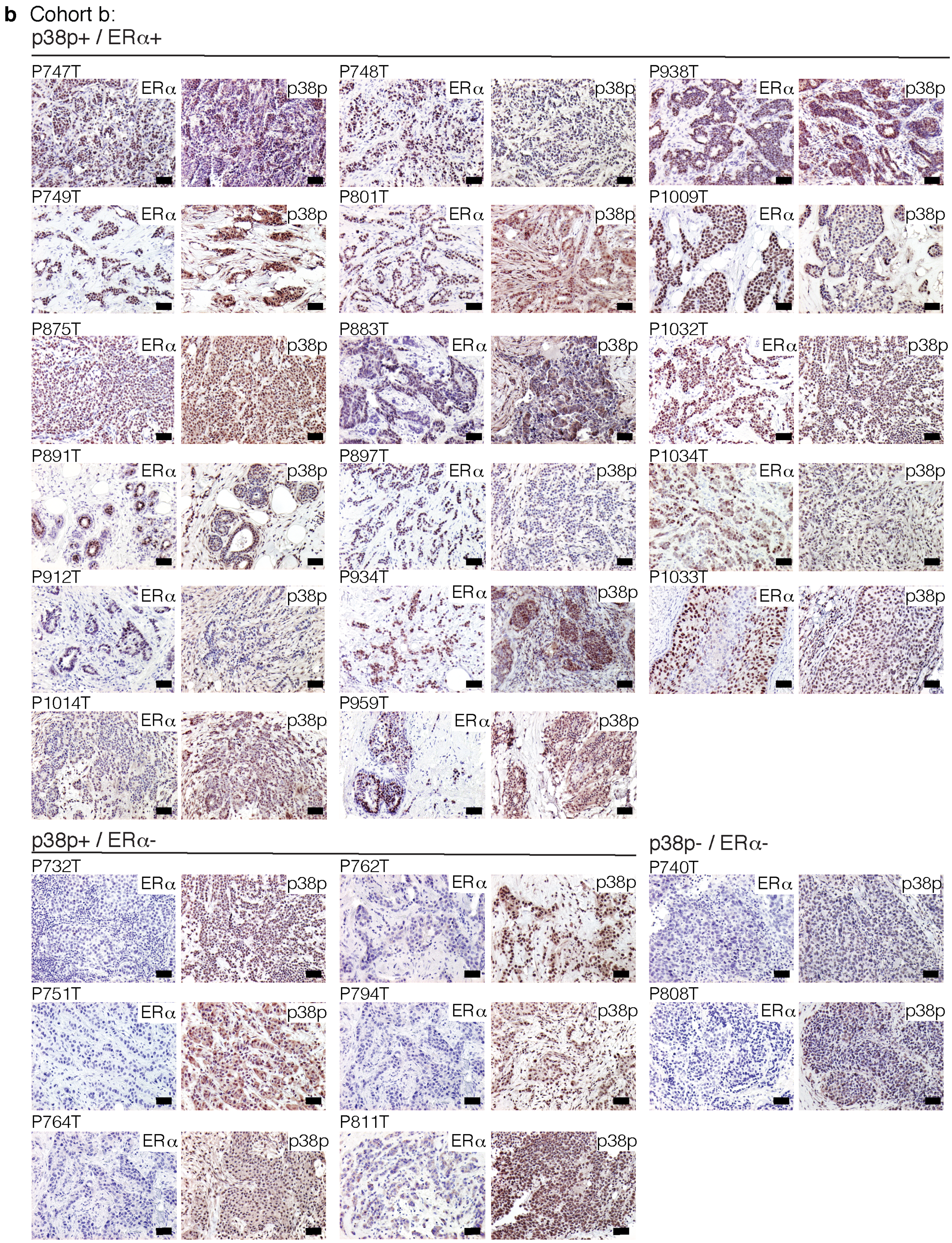
b Cohort b:
p38p+ / ERα+
P747T
ERα
p38p
P748T
ERα
p38p
P938T
ERα
p38p
P749T
ERα
p38p
P801T
ERα
p38p
P1009T
ERα
p38p
P875T
ERα
p38p
P883T
ERα
p38p
P1032T
ERα
p38p
P891T
ERα
p38p
P897T
ERα
p38p
P1034T
ERα
p38p
P912T
ERα
p38p
P934T
ERα
p38p
P1033T
ERα
p38p
P1014T
ERα
p38p
P959T
ERα
p38p
p38p+ / ERα-
P732T
ERα
p38p
P762T
ERα
p38p
P751T
ERα
p38p
P794T
ERα
p38p
P764T
ERα
p38p
P811T
ERα
p38p
p38p- / ERα-
P740T
ERα
p38p
P808T
ERα
p38p

Supplementary Figure 7. Correlation of Phospho-38 and ERα in Breast Cancer IHC Samples. **a,** Immunohistochemical staining of consecutive slices of 16 breast tumor samples (cohort a) for phosphorylated (Thr180/Tyr182) p38 and ERα. Individual tumors were categorized into p38+ / ERα+ (n = 10), p38+ / ERα− (n = 3) and p38- / ERα− (n = 3). **b**, cohort b of 25 tumors stained for phosphorylated (Thr180/Tyr182) p38 and ERα. Individual tumors were categorized into p38+ / ERα+ (n = 17), p38+ / ERα− (n = 6) and p38- / ERα− (n = 2). Scale bar 50= μm.

Supplementary Figure 8.

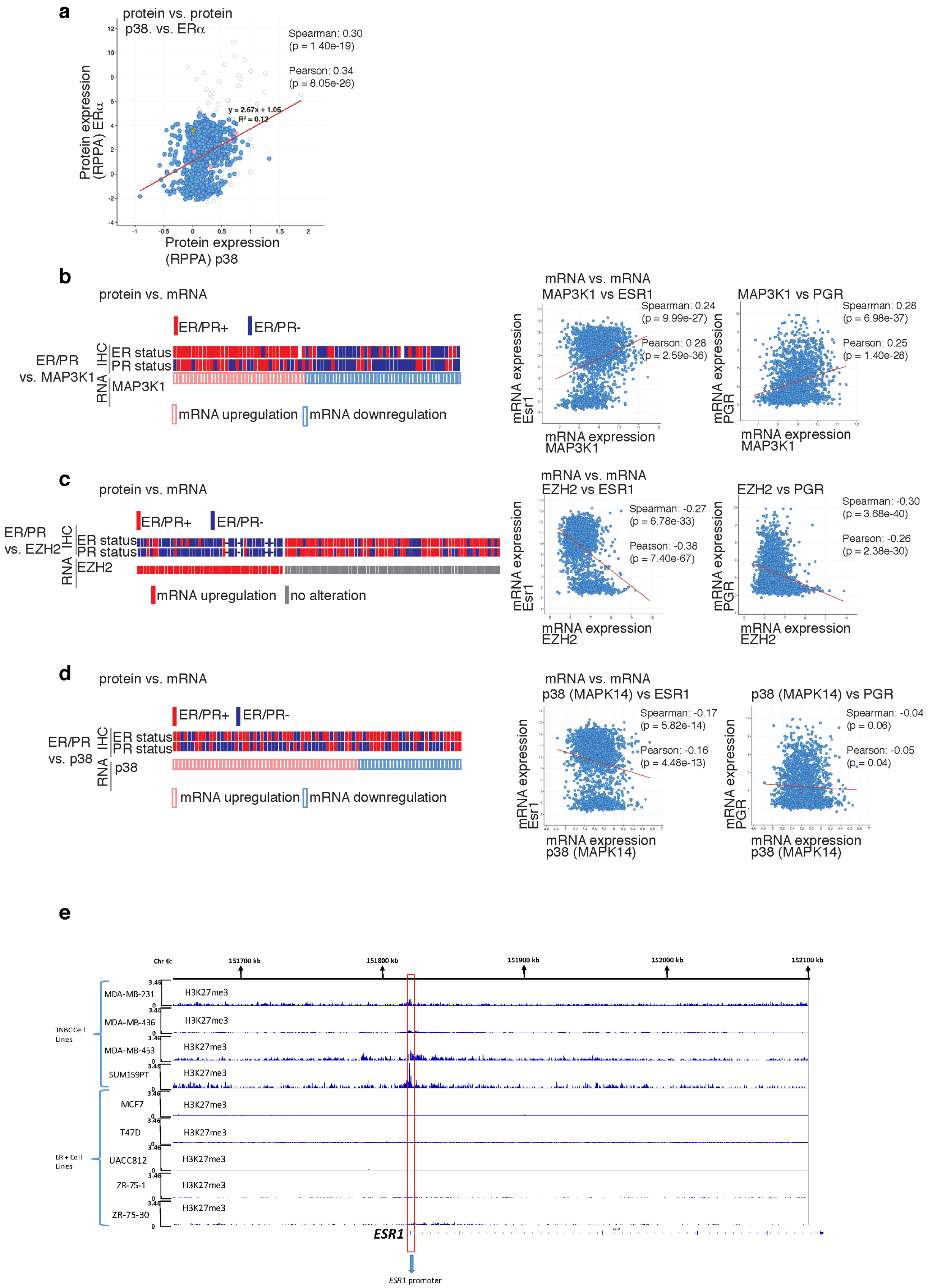

Supplementary Figure 8. Data from cBioPortal cancer genomic datasets for breast cancer. **a**, Correlation between p38 and ERα protein levels in breast cancer. Breast invasive carcinoma, TCGA, Provisional dataset for 892 RPPA in cBioPortal was examined. Spearman and Pearson correlation coefficients r and their corresponding p-values are shown (p-values were calculated with 2-sided t-test). **b**, Correlation between ERα /progesteron receptor (PR) protein (IHC) or mRNA and MAP3K1 mRNA in cBioPortal cancer genomic datasets for breast cancer. Spearman and Pearson correlation coefficients r and their corresponding p-values are shown (p-values were calculated with 2-sided t-test). **c**, Correlation between ERα/PR and EZH2 mRNA expression. **d**, ERα and p38 mRNA levels do not correlate. Spearman and Pearson correlation coefficients r and their corresponding p-values are shown (p-values were calculated with 2-sided t-test). **e**, H3K27me3 peaks at the promoter region of *Esr1* in TNBC (MDA-MB-231, MDA-MB-436, MDA-MB-453, SUM159PT) and ERα+ (MCF7, T47D, UACC812, ZR-75-1 ZR-75-30) breast cancer cell lines. Shown are ChIP-seq data from the public Cistrome database (www.cistrome.org)[3-8]. The peaks are visualized using Integrative Genomics Viewer (IGV).

Supplementary Figure 9.

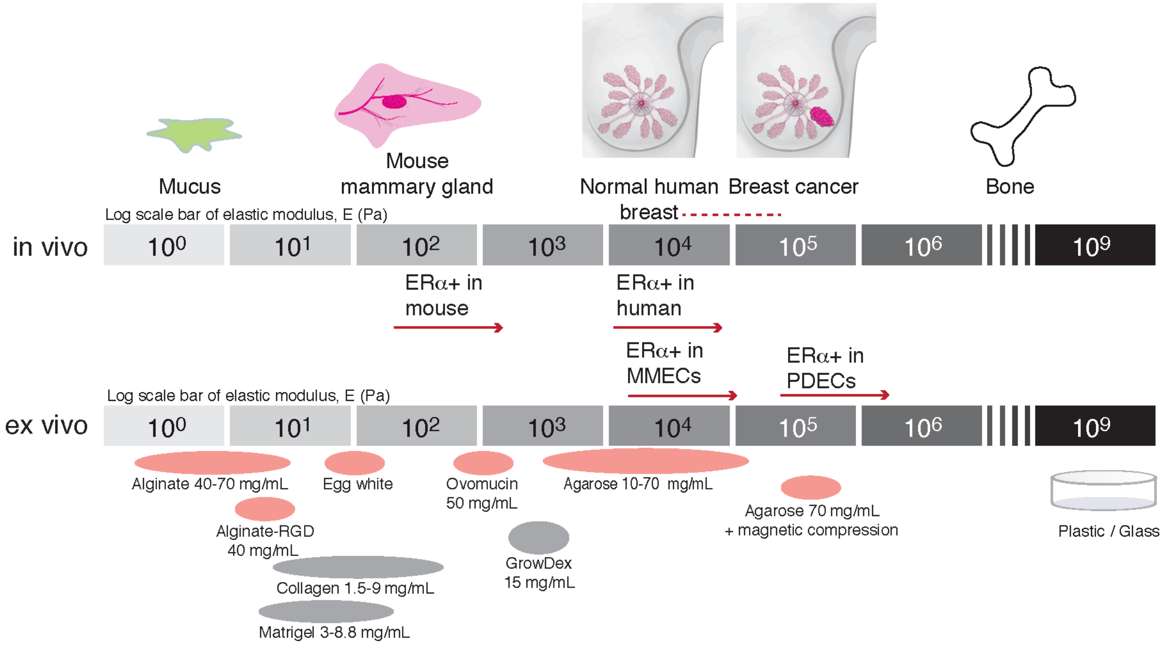

Supplementary Figure 9. Compressive Forces Impacting the Indicated Tissues and the 3D Culture Conditions Explored in this Study. The *in vivo* estimates for the breast tissue and breast cancer are from [9-14]. The *ex vivo* estimates for the explant cultures are from the present study. Grey color denotes BMx gels and red color LMx gels.

Glossary of the rheometrical terms

Stress – force (in Newtons) per unit area (in m^2). The SI unit is N/m^2 (or Pascal, Pa).

Strain –unitless parameter quantifying the extent of deformation after application of mechanical stress.

Shear Stress (σ) –the ratio of the tangential force to the cross-sectional area of the surface upon which it acts.

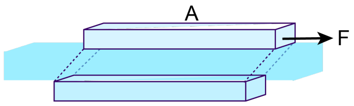

Shear Strain (γ) –unitless parameter quantifying the extent of deformation after application of shear
stress. For a cube, shear strain is ratio of lateral displacement over sample height.

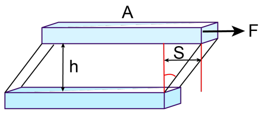

$$\gamma = \frac{S}{h}$$

Shear Modulus (G) – a constant describing a material's resistance to deformation in shear;
$G = \frac{\sigma}{\gamma}$ The SI unit is Pa.

For parallel plate geometry the shear stress (σ) and shear strain (γ) are defined as follows.

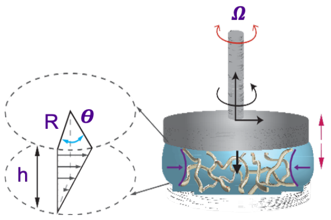

$\sigma = \frac{2}{\pi R3} M$

$\gamma = \frac{R}{h} \boldsymbol{\theta}$

R = the radius of plate
M = torque (also called as moment),
h = the gap size
θ = deflection angle (angular displacement) of the shaft

Modulus (G) is the measure of materials overall resistance to deformation. $G = \frac{strain}{Strain}$ and the SI unit is Pa.

Elastic or storage modulus (G′) – measure of energy stored during a strain cycle; under sinusoidal conditions, the part of shear stress in phase with shear strain divided by shear strain.

$$G' = \frac{\sigma}{\gamma}\cos(\boldsymbol{\theta})$$

Viscous or loss modulus (G") – measure of energy lost during a strain cycle; often expressed as the
imaginary part of the complex modulus

$$G'' = \frac{\sigma}{\gamma}\sin(\boldsymbol{\theta})$$

Young's modulus (E) is one of the most common measures of intrinsic material stiffness. In isotropic materials, E (Young´s modulus) and G` *(shear elastic modulus)* are related to each other through Poisson's ratio (ν), given by the following equation:

$$E = 2G(1+\upsilon)$$

Poisson's ratio measures the deformation in the material in a direction perpendicular to the direction of the applied force. For many common materials, Poisson's ratio is similar to that of incompressible rubber (v = 0.5). Thus, E is frequently approximated to $3G$.

Complex modulus (G*): The complex shear modulus **(G*)** is the overall resistance of the gel to the deformation and it is calculated from stress and strain amplitudes.

G* = $G' + iG''$. (where $i = (-1)^{0.5}$

Strain-stiffening: an increase in a material's elastic modulus with applied strain.

Supplementary References

1 Wadsworth, S. A. *et al.* RWJ 67657, a potent, orally active inhibitor of p38 mitogen-activated protein kinase. *J Pharmacol Exp Ther* **291**, 680-687 (1999).

2 Kumar, S., Jiang, M. S., Adams, J. L. & Lee, J. C. Pyridinylimidazole compound SB 203580 inhibits the activity but not the activation of p38 mitogen-activated protein kinase. *Biochem Biophys Res Commun* **263**, 825-831, doi:10.1006/bbrc.1999.1454 (1999).

3 Su, Y. *et al.* Somatic Cell Fusions Reveal Extensive Heterogeneity in Basal-like Breast Cancer. *Cell Rep* **11**, 1549-1563, doi:10.1016/j.celrep.2015.05.011 (2015).

4 Chaligné, R. *et al.* The inactive X chromosome is epigenetically unstable and transcriptionally labile in breast cancer. *Genome Res* **25**, 488-503, doi:10.1101/gr.185926.114 (2015).

5 Davis, C. A. *et al.* The Encyclopedia of DNA elements (ENCODE): data portal update. *Nucleic Acids Res* **46**, D794-D801, doi:10.1093/nar/gkx1081 (2018).

6 Franco, H. L. *et al.* Enhancer transcription reveals subtype-specific gene expression programs controlling breast cancer pathogenesis. *Genome Res* **28**, 159-170, doi:10.1101/gr.226019.117 (2018).

7 Zhang, G. *et al.* FOXA1 defines cancer cell specificity. *Sci Adv* **2**, e1501473, doi:10.1126/sciadv.1501473 (2016).

8 Shen, H. *et al.* Suppression of Enhancer Overactivation by a RACK7-Histone Demethylase Complex. *Cell* **165**, 331-342, doi:10.1016/j.cell.2016.02.064 (2016).

9 Wu, H. *et al.* A Preliminary Comparative Study of Young's Modulus Versus Shear Modulus in the Diagnosis of Breast Cancer. *Ultrasound Q* **35**, 88-92, doi:10.1097/RUQ.0000000000000434 (2019).

10 Ramião, N. G. *et al.* Biomechanical properties of breast tissue, a state-of-the-art review. *Biomech Model Mechanobiol* **15**, 1307-1323, doi:10.1007/s10237-016-0763-8 (2016).

11 Goliwas, K. F., Marshall, L. E., Ransaw, E. L., Berry, J. L. & Frost, A. R. A recapitulative three-dimensional model of breast carcinoma requires perfusion for multi-week growth. *J Tissue Eng* **7**, 2041731416660739, doi:10.1177/2041731416660739 (2016).

12 Paszek, M. J. *et al.* Tensional homeostasis and the malignant phenotype. *Cancer Cell* **8**, 241-254, doi:10.1016/j.ccr.2005.08.010 (2005).

13 Van Houten, E. E., Doyley, M. M., Kennedy, F. E., Weaver, J. B. & Paulsen, K. D. Initial in vivo experience with steady-state subzone-based MR elastography of the human breast. *J Magn Reson Imaging* **17**, 72-85, doi:10.1002/jmri.10232 (2003).

14 McKnight, A. L. *et al.* MR elastography of breast cancer: preliminary results. *AJR Am J Roentgenol* **178**, 1411-1417, doi:10.2214/ajr.178.6.1781411 (2002).
