# Supplementary material for: Compressive stress-mediated p38 activation required for ERα + phenotype in breast cancer
Source: Nat Commun. 2021 Nov 29;12:6967. doi: 10.1038/s41467-021-27220-9 (PMC8630031; doi:10.1038/s41467-021-27220-9)
Supplement: Supplementary file 3 — Description of Additional Supplementary Files [file 41467_2021_27220_MOESM3_ESM.pdf]

File Name: Supplementary Data 1.
Description: Histopathological and immunohistochemical parameters of the breast cancer and reduction mammoplasty samples used in the study.

File Name: Supplementary Data 2.
Description: The list of antibodies used in the study.
