# Supplementary material for: Compressive stress-mediated p38 activation required for ERα + phenotype in breast cancer
Source: Nat Commun. 2021 Nov 29;12:6967. doi: 10.1038/s41467-021-27220-9 (PMC8630031; doi:10.1038/s41467-021-27220-9)
Supplement: Supplementary file 6 — Reporting Summary [file 41467_2021_27220_MOESM6_ESM.pdf]

nature portfolio

Corresponding author(s): Juha Klefström

Last updated by author(s): 30.10.2021

Reporting Summary

Nature Portfolio wishes to improve the reproducibility of the work that we publish. This form provides structure for consistency and transparency in reporting. For further information on Nature Portfolio policies, see our Editorial Policies and the Editorial Policy Checklist.

Statistics

For all statistical analyses, confirm that the following items are present in the figure legend, table legend, main text, or Methods section.

n/a	Confirmed	
☐	☒	The exact sample size (*n*) for each experimental group/condition, given as a discrete number and unit of measurement
☐	☒	A statement on whether measurements were taken from distinct samples or whether the same sample was measured repeatedly
☐	☒	The statistical test(s) used AND whether they are one- or two-sided<br>*Only common tests should be described solely by name; describe more complex techniques in the Methods section.*
☐	☒	A description of all covariates tested
☐	☒	A description of any assumptions or corrections, such as tests of normality and adjustment for multiple comparisons
☐	☒	A full description of the statistical parameters including central tendency (e.g. means) or other basic estimates (e.g. regression coefficient) AND variation (e.g. standard deviation) or associated estimates of uncertainty (e.g. confidence intervals)
☐	☒	For null hypothesis testing, the test statistic (e.g. *F*, *t*, *r*) with confidence intervals, effect sizes, degrees of freedom and *P* value noted<br>*Give P values as exact values whenever suitable.*
☒	☐	For Bayesian analysis, information on the choice of priors and Markov chain Monte Carlo settings
☐	☒	For hierarchical and complex designs, identification of the appropriate level for tests and full reporting of outcomes
☐	☒	Estimates of effect sizes (e.g. Cohen's *d*, Pearson's *r*), indicating how they were calculated

Our web collection on statistics for biologists contains articles on many of the points above.

Software and code

Policy information about availability of computer code

Data collection	SEM images were acquired with a Zeiss Sigma VP scanning electron microscope with an acceleration voltage of 1.0 to 1.5 kV. Images of the structures were acquired using a Leica TCS SP8 CARS confocal microscope. IHC images were taken with a Leica DM LB microscope or with a Zeiss AxioImager 1.
Data analysis	Data was analysed using GraphPad PRISM 8 (Version 8.4.3). In RNA sequencing the differentially expressed genes between different groups were found using state-of-the-art statistical methods and packages, such as edgeR/DESeq2. The Gene Set Enrichment Analysis 3.0 (Broad Institute) was used to analyze the differences in the gene expression profiles. GSEA results were visualized using Cytoscape (v.3.7.2) and the enrichment map plug-in.

For manuscripts utilizing custom algorithms or software that are central to the research but not yet described in published literature, software must be made available to editors and reviewers. We strongly encourage code deposition in a community repository (e.g. GitHub). See the Nature Portfolio guidelines for submitting code & software for further information.

Data

Policy information about availability of data

All manuscripts must include a data availability statement. This statement should provide the following information, where applicable:

- Accession codes, unique identifiers, or web links for publicly available datasets
- A description of any restrictions on data availability
- For clinical datasets or third party data, please ensure that the statement adheres to our policy

The RNA sequencing data generated in this study have been deposited in Sequence Read Archive (SRA) database and are accessible through the SRA accession numbers: PRJNA663587, PRJNA663448, PRJNA663028. The BRB-RNA sequencing data generated in this study have been deposited in Sequence Read Archive (SRA)

database and are accessible through the SRA accession numbers: PRJNA775661 and PRJNA775657. Due to the nature of the consent given by the patients, we are not allowed to share the exome sequencing data in any public data repositories. The publicly available H3K27me3 ChIP-seq data from MDA-MB-231, MDA-MB-453, and SUM-159PT cell lines are available in NCBI GEO database under accession code GSE38548. The publicly available H3K27me3 ChIP-seq data from MDA-MB-436 cell line are available in NCBI GEO database under accession code GSE62907. The publicly available H3K27me3 ChIP-seq data from MCF7 cell line are available in ENCODE database under accession code ENCSR761DLU_2. The publicly available H3K27me3 ChIP-seq data from UACC812 and ZR-75-1 cell lines are available in NCBI GEO database under accession code GSE85158. The publicly available H3K27me3 ChIP-seq data from T47D cell line are available in NCBI GEO database under accession code GSE63109. The publicly available H3K27me3 ChIP-seq data from ZR-75-30 cell line are available in NCBI GEO database under accession code GSE71323. Source data are provided with this paper. The remaining data are available within the Article, Supplementary Information or Source Data file.

Field-specific reporting

Please select the one below that is the best fit for your research. If you are not sure, read the appropriate sections before making your selection.

[x] Life sciences [ ] Behavioural & social sciences [ ] Ecological, evolutionary & environmental sciences

For a reference copy of the document with all sections, see nature.com/documents/nr-reporting-summary-flat.pdf

Life sciences study design

All studies must disclose on these points even when the disclosure is negative.

Sample size	Breast cancer samples from 313 patients and 123 reduction mammoplasty samples were used in this study. For cell line and drug treatment experiments, at least 3 biological experiments were performed according to the good scientific practice. No statistical methods were used to pre-determine sample size. For ex vivo experiments all samples coming to the project were used. For in vitro experiments the n numbers were determined according to the minimal number of independent biological replicates that significantly identify an effect.
Data exclusions	No data was excluded from the analysis.
Replication	The reproducibility of the experiments was confirmed using at least three biological replicates or replicates involving multiple explants derived from different patients. The number of patients and explants used in each experiment are indicated in figures.
Randomization	Randomized studies were not included as primary cultures were generated from all reduction mammoplasty and tumor material that was received. Samples were randomly subjected to treatment or control.
Blinding	Immunofluorescent confocal samples were blinded for analysis. As potential sources of biasness were not identifiable for other assays than immunofluorescent staining the investigators were not blinded to allocation during experiments and outcome assessment.

Reporting for specific materials, systems and methods

We require information from authors about some types of materials, experimental systems and methods used in many studies. Here, indicate whether each material, system or method listed is relevant to your study. If you are not sure if a list item applies to your research, read the appropriate section before selecting a response.

Materials & experimental systems

n/a	Involved in the study	
[ ]	[x]	Antibodies
[ ]	[x]	Eukaryotic cell lines
[x]	[ ]	Palaeontology and archaeology
[ ]	[x]	Animals and other organisms
[ ]	[x]	Human research participants
[x]	[ ]	Clinical data
[x]	[ ]	Dual use research of concern

Methods

n/a	Involved in the study	
[x]	[ ]	ChIP-seq
[x]	[ ]	Flow cytometry
[x]	[ ]	MRI-based neuroimaging

Antibodies

Antibodies used	All used antibodies have been described (including catalogue number) in the methods section.
Validation	All used antibodies are commercially available and have been validated for the application by the manufacturer. The validation information is available on the the suppliers homepage. Additionally these antibodies used in this study for immunohistochemistry were further validated in the Helsinki University Hospital pathology core or in the Juha Klefstom's research laboratory by using negative and positive controls, and serial dilutions of the antibodies. Western blot and immunofluorescent antibodies were typically validated using positive and negative controls.

Eukaryotic cell lines

Policy information about cell lines

Cell line source(s)	DU4475, HCC38, BT-549, HCC1806, MCF7, T47D, MDA-MB-468, BT-20. All cell lines were obtained from ATCC.
Authentication	Only low passage cell lines (directly from ATCC) were used to avoid cross-contamination or phenotypic change upon culture. Non of the cell lines were authenticated.
Mycoplasma contamination	All cell lines, which were used in this study, were tested as mycoplasma negative.
Commonly misidentified lines (See ICLAC register)	No misidentified cell lines were used in the study.

Animals and other organisms

Policy information about studies involving animals; ARRIVE guidelines recommended for reporting animal research

Laboratory animals	Four month old female NMRI mice were maintained in a pathogen-free (SPF) facility at the University of Helsinki. The mice were maintained under standard conditions in ventilated animal cages at +23°C, 60% humidity, 12 hour dark and light cycle with standard diet and water.
Wild animals	No wild animals were used in the study
Field-collected samples	No field samples were used in the study.
Ethics oversight	All animals were covered by a license (ESAVI-2010-05551_Ym-23, KEK19-002,) approved by the National Animal Experiment Board of Finland (Eläinkoelautakunta, ELLA).

Note that full information on the approval of the study protocol must also be provided in the manuscript.

Human research participants

Policy information about studies involving human research participants

Population characteristics	Breast cancer samples were obtained from 313 breast cancer patients with primary tumor and 123 reduction mammoplasty samples from elective breast surgeries performed at the Helsinki University Central Hospital. All participants were women and the detailed histological subtypes of the tissues can be found in Supplementary Data 1.
Recruitment	Patients participated in the study by signing an informed consent form following the Declaration of Helsinki principles.
Ethics oversight	Fresh tissue was obtained from the elective breast cancer surgeries performed at the Helsinki University Central Hospital (Ethical permit: 243/13/03/02/2013/ TMK02 157 and HUS/2697/2019, approved by the Helsinki University Hospital Ethical Committee).

Note that full information on the approval of the study protocol must also be provided in the manuscript.
